# High-Sensitivity Cardiac Troponin T and the Diagnosis of Cardiovascular Disease in the Emergency Room: The Importance of Combining Cardiovascular Biomarkers with Clinical Data

**DOI:** 10.3390/jcm11133798

**Published:** 2022-06-30

**Authors:** Michele Golino, Jacopo Marazzato, Federico Blasi, Matteo Morello, Valentina Chierchia, Cristina Cadonati, Federica Matteo, Claudio Licciardello, Martina Zappa, Walter Ageno, Alberto Passi, Fabio Angeli, Roberto De Ponti

**Affiliations:** 1 Department of Medicine and Surgery, University of Insubria, 21100 Varese, Italy; j.marazzato@uninsubria.it (J.M.); fblasi@uninsubria.it (F.B.); valentina.chierchia@me.com (V.C.); federicamatteo91@gmail.com (F.M.); claudio.licci@hotmail.it (C.L.); marty-italy92@hotmail.it (M.Z.); walter.ageno@uninsubria.it (W.A.); alberto.passi@uninsubria.it (A.P.); angeli.internet@gmail.com (F.A.); roberto.deponti@uninsubria.it (R.D.P.); 2 Ospedale di Circolo, ASST Settelaghi, 21100 Varese, Italy; freshairexp@gmail.com (M.M.); cristina.cadonati@gmail.com (C.C.); 3 School of Cardiology, University of Brescia, 25121 Brescia, Italy; 4 Department of Medicine and Cardiopulmonary Rehabilitation, Maugeri Care and Research Institute, IRCCS Tradate, 21049 Tradate, Italy

**Keywords:** cardiac troponin, high sensitivity cardiac troponin, myocardial injury, acute coronary syndrome, ischemic heart disease, emergency room, myocardial ischemia, diagnosis

## Abstract

Background. Nowadays, it is still not possible to clinically distinguish whether an increase in high-sensitivity cardiac troponin (hs-cTn) values is due to myocardial injury or an acute coronary syndrome (ACS). Moreover, predictive data regarding hs-cTnT in an emergency room (ER) setting are scarce. This monocentric retrospective study aimed to improve the knowledge and interpretation of this cardiac biomarker in daily clinical practice. Methods. Consecutive adult patients presenting at the ER and hospitalized with a first abnormal hs-cTnT value (≥14 ng/L) were enrolled for 6 months. The baseline hs-cTnT value and the ensuing changes and variations were correlated with the clinical presentation and the type of diagnosis. Subsequently, multivariable models were built to assess which clinical/laboratory variables most influenced hospital admissions in the investigated population analyzed according to the final reason for hospitalization: (1) cardiovascular vs. non-cardiovascular diagnosis, and (2) ACS vs. non-ACS one. Results. A total of 4660 patients were considered, and, after a first screening, 4149 patients were enrolled. Out of 4129 patients, 1555 (37.5%) had a first hs-cTnT ≥14 ng/L, and 1007 (65%) were hospitalized with the following types of diagnosis: ACS (182; 18%), non-ACS cardiovascular disease (337; 34%) and non-cardiovascular disease (487; 48%). Higher hs-cTnT values and significant hs-cTnT variations were found in the ACS group (*p* < 0.01). The mean percentage of variation was higher in patients with ACS, intermediate in those with non-ACS cardiovascular disease, and low in those with non-cardiovascular disease (407.5%, 270.6% and 12.4%, respectively). Only syncope and CRP (OR: 0.08, 95% CI: 0.02–0.39, *p* < 0.01 and OR: 0.9988, 95% CI: 0.9979–0.9998, *p* = 0.02, respectively) or CRP (OR: 0.9948, 95% CI: 0.9908–0.9989, *p* = 0.01) and NT-proBNP (OR: 1.0002, 95% CI: 1.0000–1.0004, *p* = 0.02) were independent predictors of a cardiovascular disease diagnosis. On the other hand, only chest pain (OR: 22.91, 95% CI: 3.97–132.32, *p* < 0.01) and eGFR (OR: 1.04, 95% CI: 1.004–1.083, *p* = 0.03) were associated with the ACS diagnosis. Conclusions. Differently from the investigated biomarkers, in this study, only clinical variables predicted hospitalizations in different patients’ subgroups.

## 1. Introduction

Cardiac troponin (cTn) I and T are still today the cornerstone biomarkers for risk stratification and the early diagnosis of myocardial infarction (MI), as reported in the latest European and American guidelines [1,2,3,4,5]. Over time, the new high-sensitivity assays have improved diagnostic accuracy, being capable of detecting levels of cTn from 10 to 100 times lower than traditional tests. In this way, the high sensitivity cTn assays sped up the MI exclusion diagnostic process, thanks also to the introduction of several algorithms in the emergency room (ER) focused on its values at time 0 and after 1 or 2 h [2,6,7,8,9,10]. Furthermore, the introduction of some clinical scores has improved the interpretation of the cTn values, even if not making them completely free from misunderstandings [11,12,13,14]. Therefore, this refinement has resulted in interpretation issues both because small amounts of cTn can be detected in healthy individuals and abnormal cTn concentrations may occur in the absence of any clinical features of myocardial ischemia [15]. This latter entity, called “myocardial injury”, encompasses a broad spectrum of cardiac and non-cardiac diseases that must be dealt with when interpreting abnormal cTn values. In support of that, it has been shown that only 30 min of ischemia, without necrosis, can cause an increase in cTn [16]. However, even today, it is not possible to clinically distinguish which increases are due to which mechanisms [17]. It follows that this is not only a management problem found in an ER setting, but, at the same time, it represents a decision-making dilemma; first, because cTn elevation indicates cardiac injury without defining the cause and, second, because it classifies the patient as at high risk, requiring careful monitoring and further diagnostic investigations regardless of the final diagnosis [5]. Finally, the absence of studies on the clinical role of the different cTn release kinetics highlights the problem of interpreting its minimally altered values in myocardial injury cases [18]. To improve the knowledge and interpretation of this biomarker in daily clinical practice, in this study, we aimed to evaluate the role of high-sensitivity cardiac troponin T (hs-cTnT) and its changes in the diagnostic process performed in the ER. The secondary purpose was to investigate the utility and diagnostic predictive capacity of both the baseline hs-cTnT value and the other parameters.

## 2. Materials and Methods

### 2.1. Study Design

This was a retrospective study involving consecutive adult patients aged >18 years old presenting at the “all-comer” emergency room (ER) of the Circolo Hospital and Macchi Foundation of Varese (Italy) with at least one hs-cTnT blood assay performed. The ER is classified as a second-level ER, which has a cardiology unit with 24-h catheterization laboratory, cardiac surgery, cardiac resuscitation unit, and 24-h radiology and laboratory services. Accordingly, it manages patients with both advanced surgical and internal medical conditions. Data were searched in the electronic medical records of the hospital. Considering that the monthly number of patients whose cardiac hs-cTnT was evaluated at the ER of our hospital was around 700 and that previous studies have shown a percentage of a first abnormal value in this setting of about 30% [19], it was estimated that about 1300 patients in 6 months would have had a first abnormal hs-cTnT value; it follows that a study period of 6 months was considered sufficient for an exploratory analysis and we chose 1 January 2019–30 June 2019 as the investigated period.

After a first screening, patients who reported a first hs-cTnT value ≥14 ng/L were considered, and according to the clinical decision, they were divided into two groups: hospitalized and discharged. For the purposes of our study, we considered only hospitalized patients by dividing them according to the type of working diagnosis (the one made by the ER unblinded physician on duty according to initial information) into three groups: acute coronary syndrome (ACS, consisting of both with and without ST-elevation MI, excluding 7 cases of unstable angina), non-ACS cardiovascular disease (i.e., heart failure, chronic coronary syndrome, atrial fibrillation, tachi and bradyarrhythmia, pulmonary embolism, pericarditis, and myocarditis), and non-cardiovascular disease. A definition for ACS was drawn from current guidelines [4]. Among the ACS assessed, the ST-elevation MI diagnoses performed prior to the ER evaluation were not included since they were referred directly to the cath lab without necessarily requiring the hs-cTnT value for hospital admission.

Demographic data, cardiovascular risk factors, and comorbidities were collected from each patient as well as entry diagnosis, home drug therapy, vital parameters, and atrial fibrillation (AF) on the first ECG. Symptoms complained at ER presentation were investigated, and blood tests performed upon hospital admission were also analyzed and collected by the hospital’s central laboratory.

The baseline hs-cTnT value was correlated with the clinical presentation and the type of diagnosis; moreover, whenever possible, the percentage of the variation between the first two hs-cTnT measurements was calculated. It was considered significant if: >20%, when the first hs-cTnT measurement was ≥14 ng/L; >50%, when the value of the first hs-cTnT measurement was <14 ng/L [20]. Subsequently, the diagnostic predictive value of hs-cTnT and other clinical-biochemical parameters was also evaluated by building 4 multivariable models to assess which clinical/laboratory variables influenced diagnosis, as follows: (1) clinical and biochemical model without hs-cTnT and NT-proBNP (base model); (2) addition of hs-cTnT to the base model; (3) addition of NT-proBNP to the base model; and (4) addition both hs-cTnT and NT-proBNP to the base models. Furthermore, to investigate which of these variables underpinned hospitalizations in the appraised population, two different study groups were identified according to the clinical reason for hospital admission (1) cardiovascular vs. non-cardiovascular diagnosis group, and (2) ACS vs. non-ACS one.

This study conforms to the Declaration of Helsinki on human research and was approved by the Ethical Committee of Insubria (study number 180 of the year 2021).

Patients were managed according to routine clinical practice. Therefore, baseline and additional hs-cTnT measurements, admission, discharge decisions as well as the type of the diagnosis were at the attending physician’s discretion.

### 2.2. Hs-cTnT Assay

The enzymatic hs-cTnT dosing system supplied to the Biochemical Laboratory of the Hospital was Elecsys Troponin T hs using a Cobas 8000/e801^®^ analyzer (Roche Diagnostics, Basel, Switzerland). This system has a mother-specific detection level of 3 ng/L, a quantification limit of 13 ng/L, and an upper limit value corresponding to the 99th percentile of 14 ng/L. When the laboratory did not provide the result because the sample was hemolyzed, the ER physician on duty was immediately informed and repeated the assay of the missing biomarker. In conclusion, only non-hemolyzed blood samples were considered for the final quantitative and qualitative analysis.

### 2.3. Statistical Analysis

Continuous variables were expressed by mean and standard deviation, or median with the interquartile range ((IQR) 25th percentile and 75th percentile); categorical variables were expressed by absolute values and percentages. For the comparison between continuous variables with normal distribution, the parametric analysis of variance (ANOVA) was used; otherwise, non-parametric ANOVA was used (Kruskal–Wallis test). For the comparison between categorical variables, the chi-square test was used. Subsequently, the uncorrected effect of each clinical-biochemical variable of interest on the different types of diagnoses was evaluated using univariate logistic regression, and the results were presented as odds ratio (OR) and relative 95% confidence interval (CI). Univariate associated variables were then selected to perform multivariate logistic regression to verify the independent effect of each after correction for all other variables of interest. Multivariable models were developed using the stepwise elimination method. The ability to distinguish between the groups of diagnoses was then evaluated by calculating the area under (AUC) the receiver operating characteristic (ROC) curve with the 95% CI. Little’s missing completely at random (MCAR) test was performed for variables with missing values not showing any systematic patterns in missing data; as a result, the Pairwise deletion method was used.

The significance level was set equal to 0.05. Statistical analysis was performed using Medcalc^®^, version 20.009 (MedCalc Software Ltd., Ostend, Belgium) and R, version 4.1.2 (R Foundation for Statistical Computing, Vienna, Austria).

## 3. Results

### 3.1. Overall Patient Population

**Figure 1:**
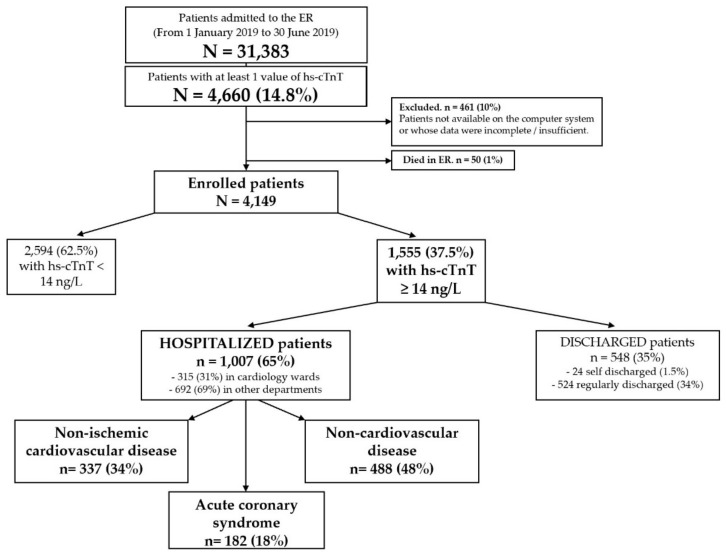
Flow-chart of the enrolled population.

From 1 January 2019 to 30 June 2019, 31,389 patients were admitted to the ER and 4660 of them (14.8%) had at least one evaluation of hs-cTnT. Of these, 50 died in the ER, while 461 were excluded from enrollment due to lack of information on the electronic medical records or because the available data were incomplete. The enrolled population was therefore 4149 patients, of which 1555 (37.5%) with a baseline hs-cTnT ≥ 14 ng/L and 2594 (62.5%) with normal hs-cTnT values (< 14 ng/L). Of the first group, 548 (35%) were discharged and 1007 (65%) were hospitalized with the following types of diagnosis: ACS (182; 18%), non-ACS cardiovascular disease (337; 34%) and non-cardiovascular disease (487; 48%). In the group with hs-cTnT < 14 ng/L, twenty-five ACS (11 non-ST elevation MI, 8 unstable angina and 3 ST-elevation MI) and 211 cases of cardiovascular disease were also recorded. Figure 1 shows the flow chart of the enrolled population.

As shown in Table 1, hospitalized patients were mostly elderly (75.6 ± 12.8 years) males (576; 57%). Prevalence of hypertension, dyslipidemia, active smoking, and diabetes was 78%, 46%, 30%, and 27%, respectively. Most of the patients had a history of chronic coronary syndrome (CCS, 30%), AF (25%), chronic kidney disease (CKD, 22%), supra-aortic trunk atheromasia (SAT, 21%) and other non-cardiovascular diseases (such as endocrinological, gastroenterological, hematological diseases, etc.). Regarding home therapies, sartans, calcium channel blockers, betablockers and inhibitors of the angiotensin I converting enzyme were the prevalent cardiovascular drugs (36%, 32%, 29% and 26%, respectively). Vital signs on hospital admission were within the normal range; however, a non-negligible proportion of patients reported AF on ECG (21%). Among signs and symptoms of presentation, dyspnea and chest pain were the most frequent (43% and 34%, respectively). Moreover, for what concerns lab results, hospitalized patients reported low hemoglobin, hematocrit, and estimated renal glomerular filtration rate (eGFR) values; on the other hand, high C-reactive protein (CRP) and N-terminal prohormone of brain natriuretic peptide (NT-proBNP) values were also shown.

Table S1 shows the demographic, clinical features, and lab results of the hospitalized patients, divided by type of diagnosis into three groups (i.e., ACS, non-ACS, and hospitalizations for reasons different from cardiovascular diseases). Male gender was predominant in the ACS group (*p* = 0.02); the same group also presented a lower mean age (*p* < 0.01). Regarding cardiovascular risk factors, a higher incidence of active smoking and familiarity with CCS was observed in the ACS group (*p* < 0.01 for both). As for comorbidities, however, a significant difference was found for all those examined (refer to Table S1 for details). Regarding home therapy, a higher prevalence of sartans, loop diuretics, MRAs, and other non-cardiovascular drugs were registered in patients with non-ACS cardiovascular disease (*p* < 0.01 for all). A significant difference was also found between groups regarding most of the vital signs, ECG, presenting signs and symptoms investigated as well as most of the lab tests, except for white blood cell count and liver function indices (refer to Table S1 for details).

### 3.2. Hs-cTnT Values

As displayed in Table 2, in the overall population, the median of the first value (40 ng/L) was nearly three times the assay cutoff (14 ng/L); moreover, among the groups considered, a higher median value was found in patients with ACS (*p* < 0.01). A second value of hs-cTnT was collected in almost half of the hospitalized patients (47%), mainly more than six hours after the first (23%), and a significant variation occurred in 30% of cases with a mean percentage of variation of 111%.

Comparing the three groups of patients, in a similar percentage of cases (almost 50%), a second hs-cTnT value was collected. Regarding the dosing time, the percentage of assays between 3 and 6 h was significantly higher in the ACS group (*p* = 0.04); on the other hand, in the other two groups, there was a non-significant higher prevalence of second assays after more than 6 h from the first. In addition, a clear difference in significant variation between the first and second hs-cTnT value was appreciated, resulting higher in the ACS group (60%) and lower in the other two (26% and 9% for non-ACS cardiovascular diseases and non-cardiovascular, respectively). Finally, the mean percentage of variation was unequivocally different between the groups, being much higher in patients with ACS, intermediate in those with non-ACS cardiovascular disease, and low in those with non-cardiovascular disease (407.5%, 270.6% and 12.4%, respectively).

### 3.3. Univariate and Multivariate Analysis: Cardiovascular Versus Non-Cardiovascular Disease

As reported in Table S2, a variety of factors were associated with the diagnosis of cardiovascular disease. In particular, the chance was more than double in active smoking patients, more than nine times in those with familiarity with CCS, more than five times in patients who complained of chest pain or palpitations, one and a half times in those with a history of heart failure. Arrhythmic heart sounds on the physical admission exam, AF on the ECG, diastolic blood pressure (DBP), SpO2, the increased values of hemoglobin, hematocrit and NT-proBNP were other factors associated with an increased chance of cardiovascular disease (refer to Table S2 for ORs, CIs, and *p*-values). Finally, a slight association was shown with increased values of the first hs-cTnT assay (OR: 1.003, 95% CI: 1.002–1.004, *p* < 0.01) or with the mean percentage of change (OR: 1.006, 95% CI: 1.003–1.009, *p* < 0.01); however, the significant variation between the first and second hs-cTnT values more than doubled the chance of cardiovascular disease (OR: 2.62, 95% CI: 1.72–3.99, *p* < 0.01).

The results of the multivariable models, including the variables that tested positive in the univariable analysis, are shown in Table 3**.** In the so-called “base model” (i.e., the one without including hs-cTnT and NT-proBNP) and in the one with hs-cTnT, only the history of syncope and CRP were associated with hospital admission with the diagnosis of cardiovascular disease. On the other hand, when NT-proBNP or both NT-proBNP and hs-cTnT were added to the “base model”, only CRP and NT-proBNP remained predictive of this diagnosis (Table 3).

Finally, regarding the ROC curve analysis, all four models had the same moderate ability to distinguish between cardiovascular and non-cardiovascular diagnosis (0.70 < AUC ≤ 0.90 and *p* < 0.01 for all), although it was slightly greater for that with the addition of NT-proBNP alone or with both NT-proBNP and hs-cTnT (AUC 0.81 for both models vs. AUC 0.76 for the other two models—Figure S1).

### 3.4. Univariate and Multivariate Analysis: Acute Coronary Syndrome (ACS) vs. Non-ACS Cardiovascular Disease

As displayed in Table S3, almost all the variables showed a significant association. In particular, the chance was more than double in active smoking patients and triple in those with familiarity with CCS. Finally, a slight association was shown with increased values of the first hs-cTnT assay (OR: 1.003, 95% CI: 1.002–1.004, *p* < 0.01) or with the mean percentage of change (OR: 1.0015, 95% CI: 1.0005–1.0025, *p* < 0.01); however, the significant variation between the first and second hs-cTnT values more than quadrupled the chance of ACS diagnosis (OR: 4.25, 95% CI: 2.49–7.27, *p* < 0.01).

The results of the multivariate models are shown in Table 4. After adjustment for different clinical variables and the investigated biomarkers, history of chest pain and eGFR values were the only independent predictors of the diagnosis of ACS in the assessed multivariable models. Interestingly, the addition of NT-proBNP or both NT-proBNP and hs-cTnT to the “base model” strengthened the association of chest pain and eGFR to the ACS diagnosis, albeit with a different weight (OR: 22.91, 95% CI: 3.97–132.32, *p* < 0.01 and OR: 1.04, 95% CI: 1.004–1.083, *p* = 0.03, respectively). Furthermore, all four models had the same moderate accuracy in predicting ACS diagnosis (0.70 < AUC ≤ 0.90 and *p* < 0.01 for all), although it was once again slightly greater for that with the addition of NT-proBNP or with both NT-proBNP and hs-cTnT (AUC 0.88 for both models—Figure S2).

## 4. Discussion

The main purpose of our study was to evaluate the role of hs-cTnT and its changes in the diagnostic process performed in the ER.

First, our data showed that in the overall patient population, 62.5% of patients had a first normal hs-cTnT value, and quite interestingly, roughly one third of those with abnormal values were discharged from the ER. Moreover, most of the patients with abnormal hs-cTnI values were hospitalized with a diagnosis that was, in nearly half of cases, a non-cardiovascular one. These data would suggest that other implied factors (i.e., clinical, laboratory and instrumental ones) would overpower abnormal hs-cTnT values in the performed clinical diagnosis affecting the decision whether to hospitalize a patient referred to the ER or not.

As to the hs-cTnT values and variations, the median value was 40 ng/L for all hospitalized patients, which is almost three times higher than the specific cutoff. Moreover, even if the highest first value was found in ACS patients, both remaining groups had hs-cTnT values more than double the specific cutoff. These data support that only one hs-cTnT value >14 ng/L does not allow a clear distinction between the three diagnosis groups. Furthermore, the ACS group had the highest percentage of patients with a significant variation between the first and second hs-cTnT value (60%); despite this, it should be emphasized that the percentage of the other two groups was not low at all (26% and 9%). Regarding the best cutoff to define a rise and fall of the cTn >99th percentile as significant, in clinical practice, the value ≥20% is used; despite this, the few studies conducted on the subject have never fully answered this question, and the only conclusion that has emerged is that the greater the delta, the greater the specificity towards the diagnosis of ACS and the risk assessment [21,22]. In the wake of this lack of solid evidence, we calculated the mean percentage of variation between the first and second hs-cTnT value, and we found an important significant difference between the groups: in fact, it was very low in patients diagnosed with a non-cardiovascular disease, very high in the ACS group and intermediate in the remainder. Regarding these data, the finding of very different values could bring new evidence in favor of the fact that the release of cTn in non-cardiovascular diseases as well as in non-ischemic cardiovascular diseases is characteristic of the so-called “myocardial injury” and different from that during necrosis; in the first case, in fact, having hypothesized a release of cytoplasmic cTn, but not that linked to myofibrils, there is a lower release peak (calculated in percentage from the difference between the second and first value divided by the second value) and half-life compared to those during necrosis [16,23,24,25]. The different kinetic effects could be the basis for and justify the different results between the groups; moreover, although there is no validated absolute cutoff to exclude or confirm an ACS, we think that its role could be further investigated, especially in an ER setting.

Considering hospitalization for cardiovascular disease, only the presence of syncope and higher CRP values remained inversely predictive. On the other hand, considering ACS diagnosis, only chest pain and higher eGFR values were independent predictors but with an important weight difference in favor of the first. This confirmed that the symptoms had more importance than the biochemical data for the final clinical decision.

In the literature, two studies also showed that the set of clinical-instrumental variables had the best capabilities of predicting an ACS diagnosis, although no data regarding cTn were reported [26,27]. On the other hand, other studies aimed to evaluate which factors were associated with an increase in hs-cTnT value and for this reason they differ from ours [28,29]. To the best of our knowledge, only Alcalai et al. [30] attempted to determine which were the clinical-laboratory predictors of ACS in the presence of abnormal hs-cTnT values, and for these characteristics, our study may be partially similar. Among the predictors of ACS, age between 40 and 70 years, history of high blood pressure or ischemic disease, normal renal function, and cTnT levels above 1 ng/mL were the most significant. However, more than half of patients with abnormal cTnT values in this study received a diagnosis of ACS, and there was no distinction between patients with non-ischemic cardiological disease and non-cardiological disease. Another difference is that the authors also reported that among patients with non-ischemic cTnT elevation, survival was lower. Due to a lack of data in our study, it was not possible to make a comparison on this aspect. We can therefore affirm how difficult it is to compare our results with those of other studies since the criteria for selecting patients, the methodology, the purposes, and the organization model of the health system can be clearly different. On the other hand, this shows the clinical importance of our study.

## 5. Limitations

Due to the absence of randomization and a control group, as well as the retrospective observational nature of the study, the presence of a selection bias regarding troponin measurement and admission to the hospital was possible in the population studied.

It was not possible to integrate all the clinical information for each patient (recall bias); chest pain characteristics were not reported for most patients. Moreover, no patient follow-up was performed due to the purely retrospective nature of the study and the lack of purpose for medium to long-term prognosis.

When two different diseases coexisted in the same patient and had a mutual cause-effect relationship, the main diagnosis of hospitalization indicated in the ER report was considered to classify cardiovascular or non-cardiovascular diseases. This could be inaccurate if data from subsequent hospitalization were taken into account, but this was not the aim of our study.

The time of the first dosage of hs-cTnT with respect to the onset of symptoms or access to the ER was very variable. Moreover, the type of diagnosis and the clinical decisions were affected by the variability in the ER medical staff. The number of hemolyzed samples was not recorded, and the hemolysis index was not calculated.

Another important limitation is the lack of a validation cohort. This certainly represents a stimulus for future studies or external independent validation.

No further analyzes were performed on the hs-cTnT data (after the first dosage) of discharged patients; this certainly represents a starting point for future studies.

Finally, an assessment of the direct and indirect costs associated with the use, the result, and significant changes of hs-cTnT could be performed subsequently.

## 6. Conclusions

This study highlights that hs-cTnT is widely used in an ER setting, and the real difference between the diagnoses is made by the mean percentage of variation, giving us a greater understanding when it comes to a myocardial injury.

In our population, hs-cTnT alone was not able to predict a cardiovascular disease or even an ACS. On the other hand, NT-proBNP was slightly more predictive than hs-cTnT for cardiovascular disease but not for ACS. Finally, chest pain proved to be the strongest predictor of ACS.

In conclusion, we support that abnormal hs-cTnT values must be evaluated with symptoms, the patient’s personal history, laboratory tests and other clinical-instrumental factors to make the most correct diagnosis. Further larger studies combining detailed clinical data with hs-cTnT concentrations in an ER setting (e.g., multicentre studies) or using more advanced technologies (e.g., machine learning) could improve knowledge in this field and refine the use of hs-cTnT.

## Figures and Tables

**Table 1:** Demographic, clinical features and lab results of the investigated patient population.

	Hospitalized Patients (N = 1007)
Male, n (%)	576 (57%)
Age (years, M ± SD)	75.6 ± 12.8
**Cardiovascular risk factors**	
Hypertension, n (%)	736 (78%)
Dyslipidemia, n (%)	385 (46%)
Active smoking, n (%)	168 (30%)
Familiarity with CCS, n (%)	59 (12%)
Diabetes, n (%)	262 (27%)
**Comorbidities**	
CCS, n (%)	280 (30%)
SAT atheromasia, n (%)	129 (21%)
Previous HF, n (%)	150 (17%)
Previous AF, n (%)	231 (25%)
CKD, n (%)	209 (22%)
COPD, n (%)	172 (18%)
Other comorbidities, n (%)	607 (67%)
**Home therapies**	
ACE-i, n (%)	227 (26%)
Sartans, n (%)	320 (36%)
Betablockers, n (%)	254 (29%)
Calcium channel blockers, n (%)	276 (32%)
Loop diuretics, n (%)	210 (24%)
MRAs, n (%)	180 (20%)
NOA/OAT, n (%)	222 (25%)
ASA/DAPT, n (%)	136 (14%)
Thiazide diuretics, n (%)	28 (3%)
Other non-cardiovascular drugs, n (%)	257 (30%)
**Vital parameters** **(median, 25°–75° percentile)**	
SBP, mmHg	130.0 (115.0–150.0)
DBP, mmHg	78.0 (65.3–89.8)
HR, bpm	81.0 (70.0–100.0)
SpO2, %	97.0 (93.0–98.0)
BT, °C	36.4 (36.0–37.2)
Rhythmic/arrhythmic heart sounds, n (%)	575 (82%)/123 (18%)
ECG: AF, n (%)	101 (21%)
**Signs and symptoms of presentation**	
Chest pain, n (%)	335 (34%)
Dyspnea, n (%)	429 (43%)
Epigastralgia, n (%)	74 (7%)
Presyncope, n (%)	37 (4%)
Syncope, n (%)	104 (10%)
Peripheral edema, n (%)	119 (12%)
Palpitations, n (%)	53 (5%)
Other non-cardiovascular symptoms, n (%)	411 (41%)
**Laboratory tests** **(Median, 25°–75° percentile)**	
**CBC**	
White blood cells, 10^6^/L	9420.0 (7242.5–12,707.5)
Hematocrit, %	38.2 (35.0–42.0)
Hb, g/dL	12.8 (11.4–14.3)
Platelets count, 10^9^/L	222.0 (176.0–281.0)
CRP, mg/L	10.3 (3.0–42.9)
INR	1.1 (1.0–1.2)
eGFR (CKD-EPI), mL/min/1.73 m^2^	57.0 (37.0–76.0)
Creatinine, mg/dL	1.2 (0.9–1.6)
Urea, mg/dL	50.0 (39.0–74.3)
AST, U/L	27.0 (21.0–38.0)
ALT, U/L	22.0 (16.0–34.0)
CPK, U/L	92.5 (55.0–158.0)
NT-proBNP, ng/L	2382.0 (754.3–6382.0)

Legend. N = number; M = mean; SD = standard deviation; CCS = chronic coronary syndromes; SAT = supra-aortic trunk; CKD = chronic kidney disease; COPD = chronic obstructive pulmonary disease; HF = heart failure; AF= atrial fibrillation; ACE-I = inhibitors of the angiotensin I converting enzyme; MRAs = mineralocorticoid receptor antagonists; NOA/OAT = new oral anticoagulants/oral anticoagulant therapy; ASA/DAPT = acetylsalicylic acid/double antiplatelet therapy; SBP = systolic blood pressure; DBP = diastolic blood pressure; HR = heart rate; SpO2 = peripheral oxygen saturation; BT = body temperature; AF = atrial fibrillation; CBC = complete blood count; Hb = hemoglobin; CRP = C-reactive protein; INR = international normalized ratio; eGFR = estimated glomerular filtration rate; CKD-EPI = Chronic Kidney Disease Epidemiology Collaboration; AST = aspartate transaminase; ALT = alanine transaminase; CPK = creatinephosphokinase; NT-proBNP = N-terminal pro-B-type natriuretic peptide.

**Table 2:** Analysis of hs-cTnT values in the overall population and in the three groups of hospitalized patients.

	Hospitalized Patients (N = 1007)	Acute Coronary Syndrome (ACS) (N = 182)	Non-ACS Cardiovascular Disease (N = 337)	Non Cardiovascular Disease (N = 488)	*p* Value
First hs-cTnT value, ng/L median (25°–75°)	40 (23–82)	74 (33–266)	42 (25–76)	33 (21–67)	<0.01
Second hs-cTnT value: overall evaluation (n, %)	476, 47	96, 53	163, 48	217, 44	0.14
at 1–3 h (n, %)	55, 6	16, 9	16, 5	23, 5	0.09
at 3–6 h (n, %)	194, 19	45, 25	69, 20	80, 16	0.04
at >6 h (n, %)	57, 23	35, 19	78, 23	114, 23	0.49
Significant ∆ between 2nd and 1st hs-cTNT values (n, %) *	142, 30	58, 60	42, 26	42, 9	<0.01
∆ % (mean) **	111	407.5	270.6	12.4	<0.01

Legend. Time 0 = defined as the time of the first troponin T value. H = hour; * Significant change defined as II value> 50% of the first if it was <14 ng/L; otherwise> 20%. ** ∆% = (Second hs-cTnT value − First hs-cTnT value)/First hs-cTnT value × 100.

**Table 3:** Multivariable models—cardiovascular versus non-cardiovascular disease.

	Odds Ratio	95% CI	*p*	AUC	95% CI	*p*
**«Base model»**						
Sincope	0.08	0.02–0.39	<0.01	0.76	0.67–0.84	<0.01
CRP	0.9988	0.9979–0.9998	0.02			
**NT-proBNP added to the** **«Base model»**						
CRP	0.9948	0.9908–0.9989	0.01	0.81	0.69–0.90	<0.01
NT-proBNP	1.0002	1.0000–1.0004	0.02			
**Firs hs-cTnT value added to the** **«Base model»**						
Sincope	0.08	0.02–0.39	<0.01	0.76	0.67–0.84	<0.01
CRP	0.9988	0.9979–0.9998	0.02			
**First hs-cTnT value + NT-proBNP added to the «Base model»**						
CRP	0.9948	0.9908–0.9989	0.01	0.81	0.69–0.90	<0.01
NT-proBNP	1.0002	1.0000–1.0004	0.02			

Legend. CI = confidence interval; ROC = receiver operating characteristic; AUC = area under the curve ROC; CRP = C-reactive protein; NT-proBNP = N-terminal fragment of the brain natriuretic propeptide; hs-cTnT = high-sensitivity cardiac troponin T. “Base model” = multivariable model without hs-cTnT and NT-proBNP.

**Table 4:** Multivariable models—ACS versus Non-ACS cardiovascular disease.

	Odds Ratio	95% CI	*p*	AUC	95% CI	*p*
**«Base model»**						
Chest pain	10.67	3.09–36.84	<0.01	0.81	0.70–0.89	<0.01
eGFR (CKD-EPI)	1.03	1.003–1.06	0.03			
**NT-proBNP added to the «Base model»**						
Chest pain	22.91	3.97–132.32	<0.01	0.88	0.75–0.95	<0.01
eGFR (CKD-EPI)	1.04	1.004–1.083	0.03			
**Firs hs-cTnT value added to the «Base model»**						
Chest pain	10.67	3.09–36.84	<0.01	0.81	0.70–0.89	<0.01
eGFR (CKD-EPI)	1.03	1.003–1.06	0.03			
**First hs-cTnT value + NT-proBNP added to the «Base model»**						
Chest pain	22.91	3.97–132.32	<0.01	0.88	0.75–0.95	<0.01
eGFR (CKD-EPI)	1.04	1.004–1.083	0.03			

Legend. CI = confidence interval; ROC = receiver operating characteristic; AUC = area under the curve ROC; eGFR = estimated glomerular filtration rate; CKD-EPI = chronic kidney disease epidemiology collaboration; NT-proBNP = N-terminal fragment of the brain natriuretic propeptide; hs-cTnT = high-sensitivity cardiac troponin T. “Base model” = multivariable model without hs-cTnT and NT-proBNP.

## Data Availability

The data presented in this study are available on request from the corresponding author. The data are not publicly available due to privacy and ethical reasons.

## Supplementary Materials

The following supporting information can be downloaded at: https://www.mdpi.com/article/10.3390/jcm11133798/s1, Figure S1. ROC curves of the four multivariable models—Cardiovascular vs. NON cardiovascular disease; Figure S2. ROC curves of the four multivariable models—ACS versus Non-ACS cardiovascular disease; Table S1. Home therapies of the three groups of hospitalized patients; Table S2. Vital signs, ECG, presenting signs and symptoms of the three groups of hospitalized patients.
