# Supplementary material for: High-Sensitivity Cardiac Troponin T and the Diagnosis of Cardiovascular Disease in the Emergency Room: The Importance of Combining Cardiovascular Biomarkers with Clinical Data
Source: J Clin Med. 2022 Jun 30;11(13):3798. doi: 10.3390/jcm11133798 (PMC9267782; doi:10.3390/jcm11133798)
Supplement: Supplementary file 1 [file jcm-11-03798-s001.zip › jcm-1763563-supplementary.pdf]

Table S1. Demographic, clinical features and lab results of the three groups of hospitalized patients.

	Acute coronary syndrome (ACS) (N = 182)	Non-ACS cardiovascular disease (N = 337)	Non cardiovascular disease (N = 488)	p value
Male, n (%)	121 (66%)	188 (56%)	267 (55%)	**0.02**
Age (years, M ± SD)	69.4 ± 12.7	76.0 ± 13.0	77.6 ± 11.9	**<0.01**
**Cardiovascular risk factors**				
Hypertension, n (%)	122 (71%)	258 (79%)	356 (79%)	0.07
Dyslipidemia, n (%)	67 (43%)	142 (49%)	176 (44%)	0.34
Active smoking, n (%)	58 (46%)	54 (30%)	57 (22%)	**<0.01**
Familiarity with CCS, n (%)	33 (30%)	20 (13%)	6 (3%)	**<0.01**
Diabetes, n (%)	43 (25%)	103 (32%)	116 (25%)	0.1
**Comorbidities**				
CCS, n (%)	42 (25%)	114 (36%)	124 (28%)	**0.01**
SAT atheromasia, n (%)	15 (13%)	49 (25%)	65 (22%)	**0.03**
Previous HF, n (%)	10 (6%)	81 (28%)	60 (14%)	**<0.01**
Previous AF, n (%)	14 (8%)	104 (36%)	113 (25%)	**<0.01**
CKD, n (%)	19 (11%)	88 (28%)	102 (23%)	**<0.01**
COPD, n (%)	15 (9%)	57 (18%)	100 (22%)	**<0.01**
Other comorbidities, n (%)	89 (56%)	214 (70%)	306 (69%)	**<0.01**
**Home therapies**				
ACE-i, n (%)	38 (24%)	89 (29%)	100 (24%)	0.20
Sartans, n (%)	43 (27%)	125 (42%)	152 (36%)	**<0.01**
Betablockers, n (%)	36 (22%)	95 (32%)	123 (29%)	0.10
Calcium channel blockers, n (%)	21 (13%)	102 (34%)	153 (37%)	**<0.01**
Loop diuretics, n (%)	21 (13%)	101 (34%)	88 (21%)	**<0.01**
MRAs, n (%)	10 (6%)	84 (28%)	86 (21%)	**<0.01**
NOA/OAT, n (%)	36 (23%)	83 (28%)	103 (25%)	0.38
ASA/DAPT, n (%)	27 (15%)	50 (15%)	59 (13%)	0.57
Thiazide diuretics, n (%)	4 (2%)	8 (2%)	16 (3%)	0.61
Other non-cardiovascular drugs, n (%)	46 (29%)	111 (37%)	21 (5%)	**<0.01**
**Vital parameters (median, 25° - 75° percentile)**				
SBP, mmHg	140.0 (125.0 – 150.0)	130.0 (112.0 – 150.0)	130.0 (113.8 – 150.0)	**<0.01**
DBP, mmHg	80.0 (72.0 – 90.0)	80.0 (65.0 – 90.0)	75.0 (62.3 – 84.0)	**<0.01**
HR, bpm	80.0 (66.3 – 90.0)	90.0 (71.5 – 110.0)	80.0 (69.5 – 96.3)	**<0.01**
SpO_2, %	98.0 (95.0 – 99.0)	97.0 (93.0 – 98.0)	96.0 (93.0 – 98.0)	**<0.01**
BT, °C	36.0 (36.0 – 36.5)	36.2 (36.0 – 36.9)	36.5 (36.0 – 37.6)	**<0.01**
**Rhythmic / arrhythmic heart sounds, n (%)**	116 (92%)/10 (8%)	176 (72%)/67 (28%)	283 (86%)/46 (14%)	**<0.01**
**ECG: AF, n (%)**	16 (12%)	62 (32%)	23 (15%)	**<0.01**
**Signs and symptoms of presentation**				
Chest pain, n (%)	152 (84%)	107 (32%)	76 (16%)	**<0.01**
Dyspnea, n (%)	34 (19%)	202 (60%)	193 (40%)	**<0.01**
Epigastralgia, n (%)	14 (8%)	18 (5%)	42 (9%)	0.19
Presyncope, n (%)	3 (2%)	12 (4%)	22 (5%)	0.21
Syncope, n (%)	10 (6%)	20 (6%)	74 (15%)	**<0.01**
Peripheral edema, n (%)	9 (5%)	61 (18%)	49 (10%)	**<0.01**

Palpitations, n (%)	8 (4%)	35 (10%)	10 (2%)	**<0.01**
Other non-cardiovascular symptoms, n (%)	52 (29%)	109 (33%)	251 (52%)	**<0.01**
**Laboratory tests (median, 25° - 75° percentile)**				
**CBC**				
White blood cells, 106/L	9440.0 (7055.0 – 12127.5)	9270.0 (7160.0 – 12570.0)	9590.0 (7395.0 – 12937.5)	0.46
Hematocrit, %	40.6 (36.5 – 44.2)	38.8 (34.8 – 42.5)	37.2 (33.1 – 41.1)	**<0.01**
Hb, g/dL	13.6 (12.3 – 15.3)	12.9 (11.4 – 14.4)	12.4 (11.0 – 13.8)	**<0.01**
Platelets count, 10^9/L	228.0 (191.0 – 292.0)	215.0 (175.0 – 264.0)	222.0 (170.0 – 288.0)	0.09
**CRP, mg/L**	4.0 (1.2 – 10.7)	8.8 (2.9 – 31.9)	17.6 (3.9 – 67.1)	**<0.01**
**INR**	0.99 (0.94 – 1.06)	1.1 (0.9 – 1.3)	1.1 (1.0 – 1.2)	**<0.01**
**Renal function**				
eGFR (CKD-EPI), mL/min/1.73 m^2	71.0 (53.0 – 86.0)	53.0 (32.8 – 70.0)	54.0 (36.0 – 73.0)	**<0.01**
Creatinine, mg/dL	1.1 (0.9 – 1.3)	1.3 (1.0 – 1.8)	1.2 (0.94 – 1.7)	**<0.01**
Urea, mg/dL	44.0 (34.0 – 53.0)	55.0 (41.0 – 84.0)	53.0 (40.0 – 77.0)	**<0.01**
**Liver function**				
AST, U/L	29.0 (22.0 – 38.0)	26.0 (21.0 – 37.0)	26.0 (21.0 – 40.0)	0.18
ALT, U/L	22.0 (16.0 - 33.8)	23.0 (16.0 – 34.0)	21.0 (15.0 – 35.0)	0.54
**CPK, U/L**	126.0 (91.0 – 193.0)	87.0 (55.0 – 138.3)	85.0 (51.0 – 156.0)	**<0.01**
**NT-proBNP, ng/L**	850.0 (226.0 – 4238.5)	3893.0 (1747.0 – 9993.5)	1498.0 (537.0 – 4164.0)	**<0.01**

Legend. N= number; M= mean; SD= standard deviation; CCS= chronic coronary syndromes; SAT= supra-aortic trunk; CKD= chronic kidney disease; COPD= chronic obstructive pulmonary disease; HF= heart failure; AF= atrial fibrillation; ACE-i= inhibitors of the angiotensin I converting enzyme; MRAs= mineralocorticoid receptor antagonists; NOA/OAT= new oral anticoagulants/oral anticoagulant therapy; ASA/DAPT= acetylsalicylic acid/double antiplatelet therapy; SBP= systolic blood pressure; DBP= diastolic blood pressure; HR= heart rate; SpO2= peripheral oxygen saturation; BT= body temperature; AF= atrial fibrillation; CBC= complete blood count; Hb= hemoglobin; CRP= C-reactive protein; INR= international normalized ratio; eGFR= estimated glomerular filtration rate; CKD-EPI= Chronic Kidney Disease Epidemiology Collaboration; AST= aspartate transaminase; ALT= alanine transaminase; CPK= creatinephosphokinase; NT-proBNP= N-terminal pro-B-type natriuretic peptide.

Table S2. Univariate Analysis - Cardiovascular versus non-cardiovascular disease.

	**Odds ratio**	**95% CI**	**p value**
Male sex	1.22	0.95 - 1.56	0.12
Age	0.98	0.97 - 0.99	**<0.01**
Active smoking	2.05	1.41 - 2.98	**<0.01**
Familiarity with CCS	9.29	3.91 - 22.05	**<0.01**
CCS	1.23	0. 93 - 1.63	0.15
SAT atheromasia	0.87	0.59 – 1.28	0.48
Previous AF	0.97	0.72 – 1.31	0.84
Previous HF	1.57	1.10 – 2.24	**0.01**
CKD	0.94	0.69 – 1.28	0.71
COPD	0.61	0.44 – 0.86	**<0.01**
Sartans	1.00	0.76 – 1.33	0.96
Calcium channel blockers	0.64	0.48 - 0.85	**<0.01**
Loop diuretics	1.37	1.00 - 1.87	0.05
MRA	0.99	0.71 - 1.37	0.93
SBP	1.00	0.99 - 1.00	0.30
DBP	1.02	1.00 – 1.03	**<0.01**
HR	1.00	0.99 – 1.02	0.06
SpO_2	1.04	1.00 - 1.08	**0.03**
BT	0.998	0.997 – 0.999	**<0.01**
Arrhythmic heart sounds	1.62	1.09 – 2.42	**0.02**

EKG: AF	1.75	1.05 – 2.92	**0.03**
Chest pain	5.38	3.98 – 7.25	**<0.01**
Dyspnea	1.26	0. 98 – 1.62	0.07
Syncope	0.34	0.22 - 0.53	**<0.01**
Peripheral edema	1.39	0.95 – 2.05	0.09
Heart-pounding	4.29	2.13 – 8.64	**<0.01**
WBC	1.00	1.00 - 1.00	0.07
Hb	1.004	1.0009 – 1.007	**<0.01**
Ht	1.0009	1.0000 -1.002	**0.02**
CRP	0.9993	0.9990 - 0.9996	**<0.01**
INR	0.9988	0.9971 – 1.0005	0.16
eGFR (CKD-EPI)	1.005	0.9998 – 1.01	0.06
Creatinine	0.9996	0.9986 – 1.0006	0.42
Urea	0.999	0.996 -1.002	0.40
CPK	0.9999	0.9997 - 1.0002	0.63
NT-proBNP	1.00	1.00 - 1.00	**0.04**
**First hs-cTnT**	1.003	1.002 - 1.004	**<0.01**
**Significant variation of hs-cTnT**	2.62	1.72 – 3.99	**<0.01**
**Mean % of variation of hs-cTnT**	1.006	1.003 – 1.009	**<0.01**

Legend. CI = confidence interval; CCS = chronic coronary syndrome; SAT= supra-aortic trunk; AF= atrial fibrillation; HF = heart failure; CKD = chronic kidney disease; COPD = chronic obstructive pulmonary disease; MRA = mineralocorticoid antagonists; SBP = systolic blood pressure; DBP = diastolic blood pressure; HR= heart rate; SpO_2 = peripheral oxygen saturation; BT= body temperature; WBC = white blood cell; Hb = hemoglobin; Ht= hematocrit; CRP = C-reactive protein; eGFR= estimated glomerular filtration rate; CKD-EPI= Chronic Kidney Disease Epidemiology Collaboration; CPK= creatinephosphokinase; NT-proBNP = N-terminal fragment of the brain natriuretic propeptide; hs-cTnT = high-sensitivity cardiac troponin T.

Table S3. Univariate Analysis - Acute coronary syndrome (ACS) vs. Non-ACS cardiovascular disease.

	**Odds ratio**	**95% CI**	**p value**
Male sex	0.65	0.45 - 0.95	**0.02**
Age	0.96	0.95 - 0.98	**<0.01**
Active smoking	2.04	1.27 - 3.29	**<0.01**
Familiarity with CCS	3.00	1.61 - 5.58	**<0.01**
CCS	0.60	0.40 - 0.91	**0.01**
SAT atheromasia	0.48	0.26 - 0.89	**0.02**
Previous AF	0.19	0.10 - 0.34	**<0.01**
Previous HF	0.17	0.09 - 0.34	**<0.01**
CKD	0.35	0.20 - 0.59	**<0.01**
COPD	0.49	0.27 - 0.88	**0.01**
Sartans	0.53	0.35 - 0.80	**<0.01**
Calcium channel blockers	0.31	0.19 - 0.52	**<0.01**
Loop diuretics	0.29	0.17 - 0.49	**<0.01**
MRA	0.20	0.10 - 0.38	**<0.01**
SBP	1.009	1.001 - 1.017	**0.02**
DBP	1.018	1.005 - 1.032	**<0.01**
HR	0.98	0.97 - 0.99	**<0.01**
SpO_2	1.07	0.99 - 1.15	**0.03**
BT	0.9985	0.9966 - 1.0004	0.13
Arrhythmic heart sounds	0.22	0.11 - 0.45	**<0.01**
EKG: AF	0.28	0.16 - 0.52	**<0.01**
Chest pain	0.09	0.06 - 0.14	**<0.01**
Dyspnea	0.16	0.10 - 0.24	**<0.01**
Syncope	0.92	0.42 - 2.00	0.82

Peripheral edema	0.23	0.11 - 0.48	**<0.01**
Heart-pounding	0.39	0.18 - 0.87	**0.01**
WBC	1.0000	1.0000 - 1.0000	0.63
Hb	1.0039	1.0002 - 1.0076	**0.01**
Ht	1.0001	0.9991 - 1.0011	0.85
CRP	0.9996	0.9991 - 1.0001	0.10
INR	0.994	0.991 - 0.998	**<0.01**
eGFR	1.029	1.021 - 1.038	**<0.01**
Creatinine	0.995	0.992 - 0.998	**<0.01**
Urea	0.986	0.980 - 0.992	**<0.01**
CPK	1.0001	0.9997 - 1.0005	0.60
NT-proBNP	0.9999	0.9998 - 1.0000	**<0.01**
**First hs-cTnT**	1.003	1.002 - 1.004	**<0.01**
**Significant variation of hs-cTnT**	4.25	2.49 - 7.27	**<0.01**
**Mean % of variation of hs-cTnT**	1.0015	1.0005 to 1.0025	**<0.01**

Legend. CI = confidence interval; CCS = chronic coronary syndrome; SAT= supra-aortic trunk; AF= atrial fibrillation; HF = heart failure; CKD = chronic kidney disease; COPD = chronic obstructive pulmonary disease; MRA = mineralocorticoid antagonists; SBP = systolic blood pressure; DBP = diastolic blood pressure; HR= heart rate; SpO_2 = peripheral oxygen saturation; BT= body temperature; WBC = white blood cell; Hb = hemoglobin; Ht= hematocrit; CRP = C-reactive protein; eGFR= estimated glomerular filtration rate; CPK= creatinephosphokinase; NT-proBNP = N-terminal fragment of the brain natriuretic propeptide; hs-cTnT = high-sensitivity cardiac troponin T.

Figure S1. ROC curves of the four multivariable models – Cardiovascular vs. NON cardiovascular disease.

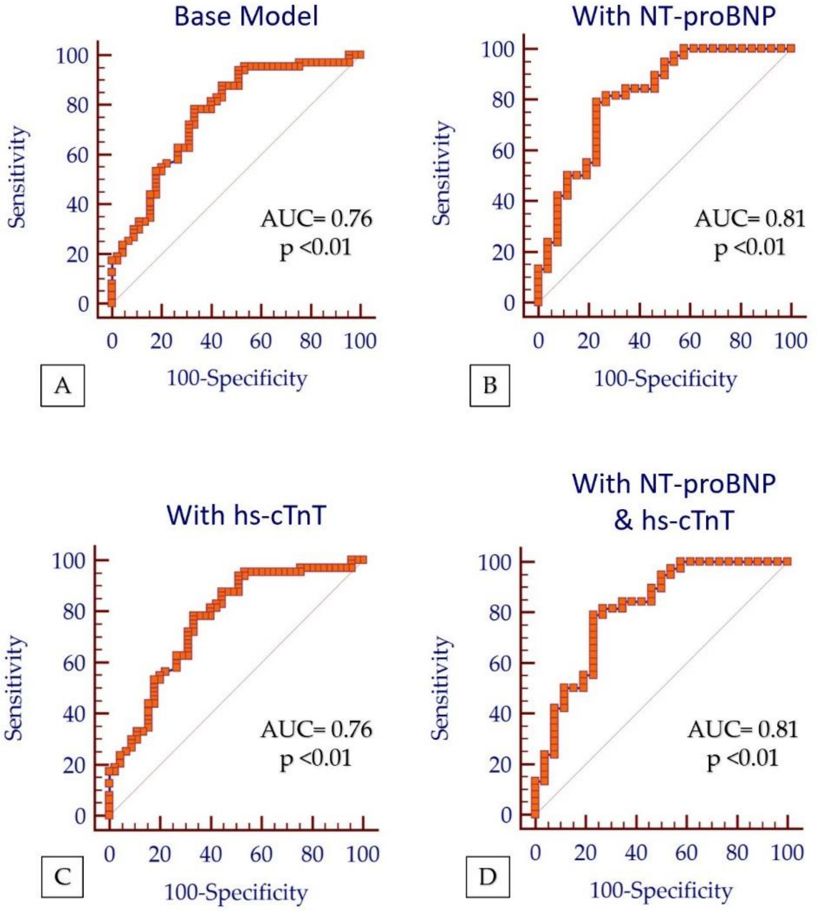

Legend. ROC= Receiver Operating Characteristic; AUC= area under the curve ROC; NT-proBNP = N-terminal fragment of the brain natriuretic propeptide; hs-cTnT = high-sensitivity cardiac troponin T. A= “Base model” = multivariable model without NT-proBNP and hs-cTnT; B= multivariable model with NT-proBNP added to the “base model”; C= multivariable model with hs-cTnT added to the “base model”; D= multivariable model with NT-proBNP and hs-cTnT added to the “base model”.

Figure S2. ROC curves of the four multivariable models – ACS versus Non-ACS cardiovascular disease.

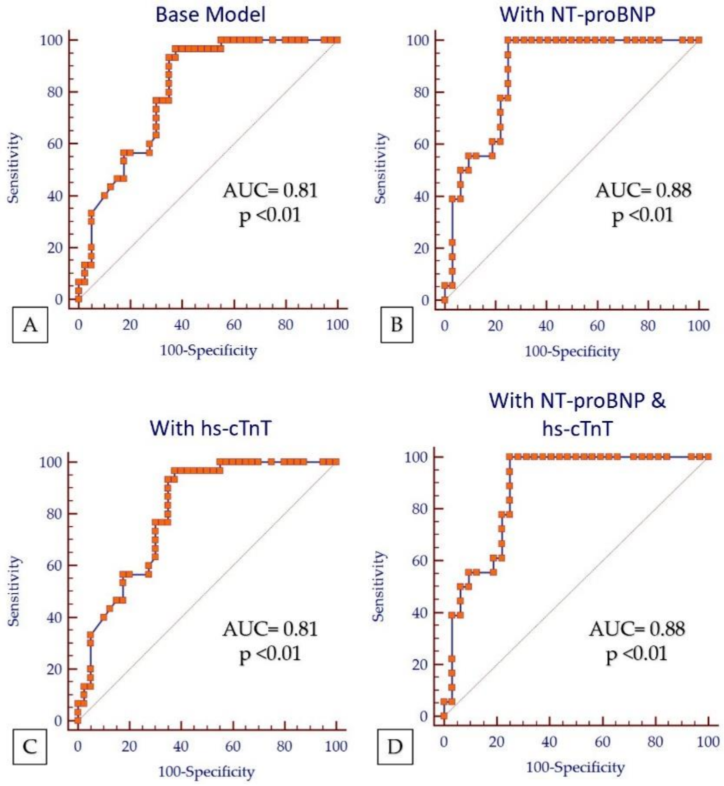

Legend. ROC= Receiver Operating Characteristic; AUC= area under the curve ROC; NT-proBNP = N-terminal fragment of the brain natriuretic propeptide; hs-cTnT = high-sensitivity cardiac troponin T. A= “Base model” = multivariable model without NT-proBNP and hs-cTnT; B= multivariable model with NT-proBNP added to the “base model”; C= multivariable model with hs-cTnT added to the “base model”; D= multivariable model with NT-proBNP and hs-cTnT added to the “base model”.
